# Dilution Reduces Sample Matrix Effects for Rapid, Direct, and Miniaturised Phenotypic Antibiotic Susceptibility Tests for Bovine Mastitis

**DOI:** 10.3390/antibiotics12091363

**Published:** 2023-08-24

**Authors:** Matthew Michael Long, Sarah Helen Needs, Alexander Daniel Edwards

**Affiliations:** 1 School of Pharmacy, University of Reading, Reading RG6 6DX, UK; m.m.long@pgr.reading.ac.uk (M.M.L.); s.h.needs@reading.ac.uk (S.H.N.); 2 Electronics and Computer Science, University of Southampton, Southampton SO17 1BJ, UK

**Keywords:** mastitis, milk, microfluidics, bacteria, AMR, resazurin

## Abstract

The time-consuming nature of current methods for detecting antimicrobial resistance (AMR) to guide mastitis treatment and for surveillance, drives innovation towards faster, easier, and more portable technology. Rapid on-farm testing could guide antibiotic selection, reducing misuse that contributes to resistance. We identify challenges that arise when developing miniaturized antibiotic susceptibility tests (AST) for rapid on-farm use directly in milk. We experimentally studied three factors: sample matrix (specifically milk or spoiled milk); the commensal bacteria found in fresh bovine milk; and result time on the performance of miniaturised AST. Microfluidic “dip-and-test” devices made from microcapillary film (MCF) were able to monitor Gram-negative bacterial growth colourimetrically even in the presence of milk and yoghurt (used to simulate spoiled milk samples), as long as this sample matrix was diluted 1:5 or more in growth medium. Growth detection kinetics using resazurin was not changed by milk at final concentrations of 20% or lower, but a significant delay was seen with yoghurt above 10%. The minimum inhibitory concentration (MIC) for ciprofloxacin and gentamicin was increased in the presence of higher concentrations of milk and yoghurt. When diluted to 1% all observed MIC were within range, indicating dilution may be sufficient to avoid milk matrix interfering with microfluidic AST. We found a median commensal cell count of 6 × 10^5^ CFU/mL across 40 healthy milk samples and tested if these bacteria could alter microfluidic AST. We found that false susceptibility may be observed at early endpoint times if testing some pathogen and commensal mixtures. However, such errors are only expected to occur when a susceptible commensal organism is present at higher cell density relative to the resistant pathogen, and this can be avoided by reading at later endpoints, leading to a trade-off between accuracy and time-to-result. We conclude that with further optimisation, and additional studies of Gram-positive organisms, it should be possible to obtain rapid results for microfluidic AST, but a trade-off is needed between time-to-result, sample dilution, and accuracy.

## 1. Introduction

Increasing evidence shows that transmission of resistant bacterial strains from dairy farms to humans and the environment is possible [1], confirming that industrial and agricultural antibiotic use is one major driver for the endless rise in antimicrobial resistance (AMR) worldwide. As well as the serious threat to human health and public safety, treatment of our dairy herds becomes ever harder as antibiotics become less effective, with overuse and misuse of antibiotics recognised as areas for improvement [1,2]. Routes of AMR from farm to humans include food production—in the case of dairy, through milk and cheese products—and via direct contact with animals [3]. Safer and more cost-effective alternative treatments, combined with innovation in diagnostics and discovering new antibiotics, have been prioritised to tackle AMR. Of highest importance is to consider some of the antibiotic classes that have been labelled as the most critically important antibiotics in dairy cattle including aminoglycosides, fluoroquinolones, third-, and fourth-generation cephalosporins and tetracyclines, as identified by the World Organisation of Animal Health (OIE) [4,5]. Equally important is maintaining appropriate testing and surveillance, so antibiotic susceptibility tests on clinical mastitis samples and bacterial isolates must be kept up-to-date to match current treatment practices and resistance profiles found in herds. Many emerging technologies are being developed that offer new ways to measure antibiotic susceptibility, which are needed to increase the frequency of testing, and ideally offer faster time-to-result.

Raw cow’s milk has been shown to contain a host of microorganisms contributing to a complex microbiota potentially affecting the efficacy of rapid testing for pathogenic mastitis-causing bacteria. It was once considered that healthy cow mammary glands are sterile and that bacteria found in milk resulted from non-sterile milk sampling, advocating precaution in aseptic milk collection [6,7]. However, studies in the microbiota of milk and the application of more advanced detection methods have overturned this assumption, identifying the frequent presence of bacterial species in a healthy milk sample, and differentiating distinct groups of normal milk samples from healthy cows by the distribution of common species present [8]. High-throughput sequencing techniques and metagenomics permit the investigation of microbial communities—termed microbiomes—of which bovine milk has become of particular interest [9,10]. The status of these microbiomes can affect outcomes after mastitis treatment [11], and conversely, antibiotic treatment can alter the microbiome [12]. With this microbial complexity in mind, some of the most common pathogen genera isolated in mastitis milk samples are *staphylococci*, *enterobacteria* and *streptococci*, together causing the majority of clinical intramammary mastitis infections [13] with mastitis-infected quarters often demonstrating a much higher bacterial load than healthy quarters [9]. However, the milk microbiome in bovine mammary quarters free from intramammary infection and inflammation, with a low milk somatic cell count, has been found to be even more diverse than that seen in quarters with clinical mastitis [14,15] presenting a problem for rapid phenotypic and direct on-farm test for the presence of mastitis infection.

With a move towards on-farm diagnostics and the need for more convenient and portable diagnostic tools, innovations are emerging such as infrared thermography (IRT) for detecting inflammation, an indicator for mastitis [16], which may offer an alternative to the California mastitis test and may also offer additional benefits such as differentiating clinical mastitis from subclinical mastitis cases. IRT detection methods are based on infrared thermal images, particularly looking at udder skin surface temperature (USST). These studies have shown a positive correlation between USST and somatic cell count (SCC), with USST increasing with SCC, with little effect of external environmental factors [17]. New phenotypic rapid testing kits, such as MastDecide (MastDecide, Quidee GmbH, Homberg, Germany), are aiming to reduce the future over-usage of antimicrobials in dairy cows with the greater aim to decrease antimicrobial resistance. Rapid detection of mastitis-causing pathogens is necessary for early detection and treatment [18], with rapid direct sample tube test systems such as MastDecide providing positive results in >14 h [19] in comparison to conventional agar growth of 24–48 h or longer to result. Current point-of-care (POC) testing is often culture-based and includes some form of a modified agar plate, petrifilm or tube-based system, such as MastDecide. However, other more recent POC devices, such as Point-of-Cow and Mastatest [20], utilise consumables and equipment that are more sophisticated and more expensive to further improve ease of use. Culture-based tests all have similar time to results of 24–48 h. Although this is fast enough to inform decision making, farmers would prefer less delay with turn-around from one milking to the next [21,22], leading to greater interest in developing rapid on-farm tests.

Our group developed a “lab-on-a-stick” concept for performing functional assays on cells including bacterial growth assays, allowing both phenotypical identification and the quantitative measurement of antibiotic susceptibility [23]. This may be extrapolated to the identification of mastitis-causing bacteria from infected milk. If adapted for rapid susceptibility testing, this technology could aid in the prescription of the correct antibiotic for treatment by rapidly indicating antibiotic resistance or susceptibility in specific strains of bacteria, improving the quality of on-farm treatment. The targeting of antibiotic treatment based on an antibiotic susceptibility test is helpful in avoiding antibiotic change due to inefficacy. However, it is important to note that any use of antibiotics in mastitis treatment will promote AMR. This technology has the potential to transport bacterial assays outside the lab and into the field for more rapid results. With this idea of on-farm treatment and diagnostics in the field in mind, we considered the use of smartphones and portable high-quality cameras to read functional cellular assays for measuring bacterial growth or death when combined with antibiotics [24,25]. The combination of low-cost microcapillary devices with smartphone imaging offers the potential for portable and field AMR detection. When phenotypically identifying bacterial species and quantifying antibiotic susceptibility, many microbiological assays rely on the identification of a colour or fluorescence change. Smartphones and digital camera modules can be used as digital sensors for colour-based bioassays, to directly capture images to digitise colourimetric and fluorescence changes [24,26]. When automated with time-lapse imaging, monitoring the growth of bacteria in different antibiotic conditions allows the identification of the correct class and concentration of antibiotics that would need to be administered to each case of mastitis. High-throughput laboratory devices have likewise been developed to provide greater flexibility in analysing the problem of AMR [27,28]. Where an automated process for microbiological techniques can improve sample time [18], the use of microfluidic technology to detect pathogens and measure AMR may offer a point-of-treatment technique. This might avoid mastitis bacteria identification in a laborious and time-consuming manner within laboratories [29]. Miniaturized devices analyse small sample volumes reducing reagent consumption and therefore potentially lowering cost [30]. Reducing space requirements compared with large agar Petri dishes may also simplify high-throughput screening even in smaller laboratories or on-farm. Simple and high-throughput microcapillary techniques can be used to test uropathogenic species of bacteria, quantify bacterial concentrations (CFU/mL) and detect resistance through minimum inhibitory concentration MIC AST [31,32], raising the possibility that point-of-care testing for mastitis infections may be possible using test volumes as small as 1 microlitre.

Here, we explore some of the challenges that must be overcome to develop miniaturized and/or rapid devices with on-farm capabilities for detecting mastitis-causing pathogens and to carry out minimum inhibitory concentrations assays for the identification of resistant bacteria directly in milk samples. We show that milk can be directly tested using a metabolic-sensitive growth indicator dye—resazurin—in low-cost microfluidic devices capable of detecting bacterial growth and determining antibiotic resistance. We explore the milk sample matrix interference on growth detection and direct antibiotic resistance testing. As fresh milk even from healthy cows contains an abundance of commensal bacteria that can interfere with the detection of mastitis pathogens, we explore if the commensal bacterial counts in healthy samples could be expected to interfere with pathogen detection. Finally, we test if false susceptibility could be observed in a sample containing a mixed population of bacteria, specifically testing simulated samples containing a susceptible commensal organism alongside a resistant pathogen.

## 2. Results

### 2.1. Outlining Challenges with Direct Rapid Field Antibiotic Susceptibility Testing

**Figure 1:**
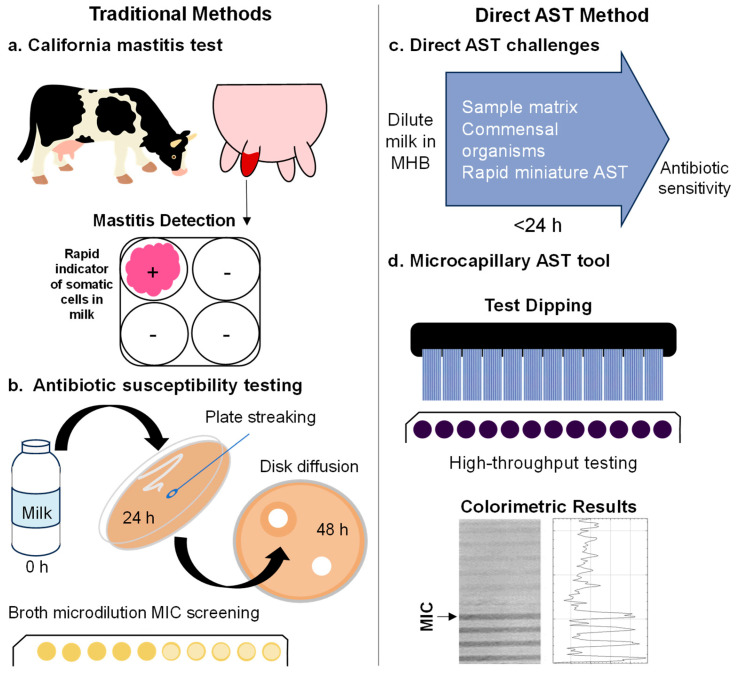
Direct microfluidic antibiotic susceptibility testing and viable cell quantitation using resazurin in cow’s milk compared to traditional methods of mastitis diagnosis and antibiotic susceptibility testing. (**a**) Traditional methods of diagnosing mastitis in cattle include the California milk test, indicating the presence of a high number of somatic cells within an infected milk sample. (**b**) Confirmation of infection and antibiotic susceptibility is carried out in lab using traditional plate count methods for enumeration of cells and disk diffusion or full MIC in well plates for antibiotic susceptibility. (**c**) Microfluidic methods described can be used to directly test milk samples, with simple dilution of milk and addition of resazurin dye. Antibiotic–coated MCF strips can then be dipped into individual milk samples. (**d**) Results allow for the enumeration of bacterial content and antibiotic susceptibility by MIC determination. Colour change can be detected by eye and confirmed by absorbance values from image analysis (ImageJ, Version 1.53t).

We examined the current diagnostic pathway to identify major challenges that need to be overcome to achieve rapid direct antibiotic susceptibility testing (AST) in milk from cows suffering from mastitis. We then experimentally tested if these challenges arise using a low-cost, scalable microfluidic platform. Currently, diagnosis relies on clinical presentation combined with tests such as somatic cell count or the California milk test (Figure 1a). Milk samples from cows with diagnosed mastitis are typically subsequently tested in laboratories using standard culture methods (Figure 1b). To be effective, direct AST methods need to avoid the milk sample matrix affecting results, avoid incorrect susceptibility scoring through interference by commensal organisms present in milk, and maintain accurate results at earlier time points and/or with smaller test volumes (Figure 1c). We explored the impact of these parameters using a low-cost and scalable microfluidic method that uses microcapillary film (MCF) test strips (Figure 1d) to kinetically monitor microbial growth using resazurin colourimetric conversion.

### 2.2. Colourimetric Microfluidic Bacterial Growth Detection in the Presence of Milk

**Figure 2:**
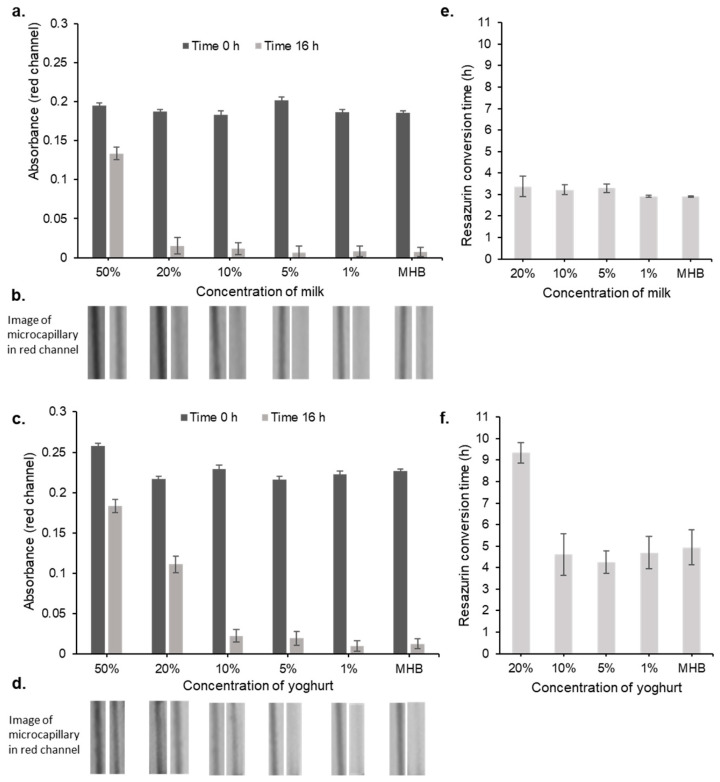
Milk sample matrix effect on absorbance of resazurin dye. Mean absorbance values and representative capillary images of resazurin dye before and after overnight incubation with *E. coli* 25,922 in the presence of milk (**a**,**b**) or (**c**,**d**) yoghurt. Bacterial growth results in resazurin conversion and decrease in absorbance. Time to resazurin conversion in the presence of milk (**e**) or yoghurt (**f**) diluted in Mueller–Hinton (MH) broth was recorded when spiked with 5 × 10^5^ CFU/mL *E.coli* 25,922. Error bars indicate ± standard error of the mean (*n* = 3).

Firstly, milk is strongly light scattering and opaque, so we tested milk matrix interference on colourimetric detection, to determine whether bacterial growth can be monitored by time-lapse imaging directly in milk. Pasteurised, homogenized supermarket milk—chosen for its low bacterial load and to improve experimental repeatability, significantly altered the visual appearance of microcapillary devices and digital images compared to Mueller–Hinton (MH) broth alone. We presumed this is due to strong light-scattering properties. Some differences in calculated absorbances within individual capillaries were detected (Figure 2a,b). Surprisingly, although visually different, with the transillumination imaging setup used here [33], clear changes in red channel absorbance still indicated that metabolic conversion of resazurin is easy to quantify colourimetrically in the presence of milk. Only the highest milk concentrations (50% final concentration) gave a large enough increase in baseline absorbance to reduce the growth detection measurement range. Equivalent concentrations of natural yoghurt were used to simulate spoiled milk and explore potential problems if poorly stored samples affected growth detection. We used yoghurt as a consistent representation of aged milk samples where microbial growth may affect composition for example by decreasing pH. Yoghurt affected colourimetric growth detection more strongly than milk, with altered absorbance values between time 0 h and 16 h in yoghurt not only at 50% but also at 20%. This suggests that at least 1:10-fold dilution may be needed, to reduce the risk of milk matrix interference, if there is any risk that samples may have spoiled prior to testing.

Time-lapse images permit growth kinetics to be estimated [34]. When growth kinetic in 20% or lower milk concentrations were compared, no delay in resazurin conversion was detected. The growth rate of *E. coli* 25299 and time to resazurin conversion was independent of the concentration of milk for 20% milk and lower, indicating that 1:5 sample dilution may be enough to avoid milk matrix interference in growth detection even at earlier endpoints for faster time-to-result (Figure 2e). However, a greater effect on time to resazurin conversion was found with yoghurt, with 20% yoghurt taking >9 h for growth to be detected (Figure 2f). With 1:10 dilution or lower, there was no delay.

### 2.3. Impact of Milk Sample Matrix on Antibiotic Minimum Inhibitory Concentration Measurement

**Figure 3:**
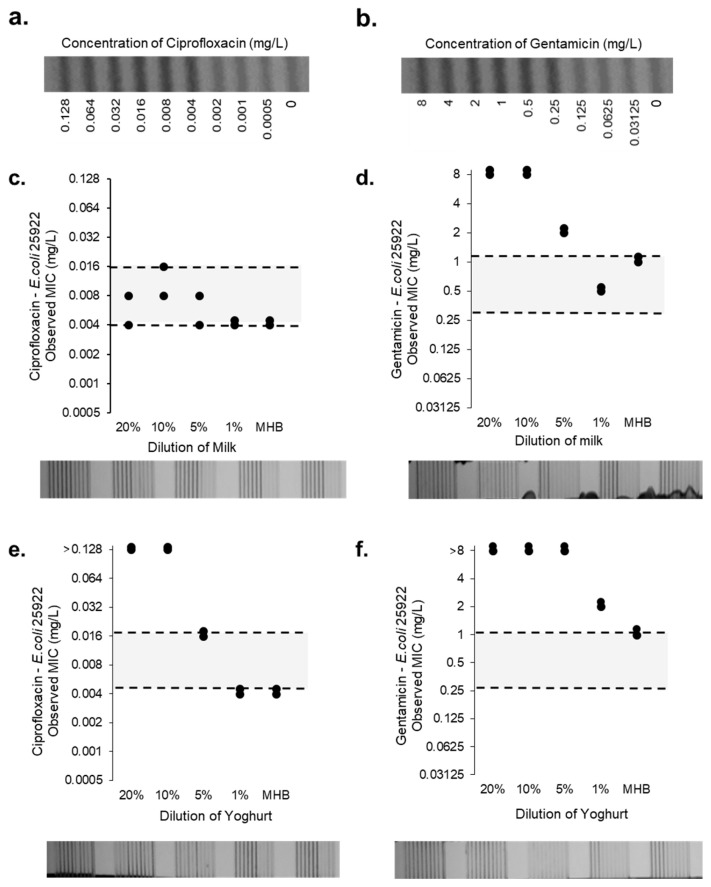
MIC determination for ciprofloxacin and gentamicin for *E. coli* 25922 QC strain in differing dilutions of milk and yoghurt in MCF (*n* = 2). MIC test strips concentrations for ciprofloxacin (**a**) and gentamicin (**b**) performed by spiking *E.coli* 25922 (5 × 10⁵ CFU/mL) in differing dilutions of pasteurised sterile (**c**,**d**) milk and (**e**,**f**) yoghurt diluted in MHB. Grey area indicates the acceptable MIC range for *E. coli* 25922. Bacterial growth results in resazurin conversion and decrease in absorbance. Images of milk and yoghurt MIC experiments are shown below. Images indicate the microcapillary test strips after overnight incubation.

Having established that growth can be detected colourimetrically in the presence of supermarket milk, we examined if milk affects the observed MIC of gentamicin and ciprofloxacin on *E. coli* 25922. At 1:5 or 1:10 dilution, milk increased the MIC of gentamicin above the expected range, but no effect was seen of 20% final concentration of milk on ciprofloxacin, with MIC values in the target range between 0.016 and 0.004 mg/L (Figure 3). Yoghurt had a greater impact on the observed MIC of *E. coli* 25922 for both ciprofloxacin and gentamicin. All concentrations of milk did not affect the MIC falling in the expected range for ciprofloxacin. This indicates that although milk may not affect some antibiotics, it may affect others, and it would be important to dilute direct milk samples to carry out MIC determination. At 1% milk the MIC for gentamicin is within the expected range (1–0.25 mg/L) suggesting it may be necessary to use dilutions of 1:100 to reduce the risk that milk affects MIC determination. Yoghurt showed a much greater effect on AST for both antibiotics. 

### 2.4. Exploring Whether Commensal Organisms in Milk Might Affect Direct Microfluidic AST

**Figure 4:**
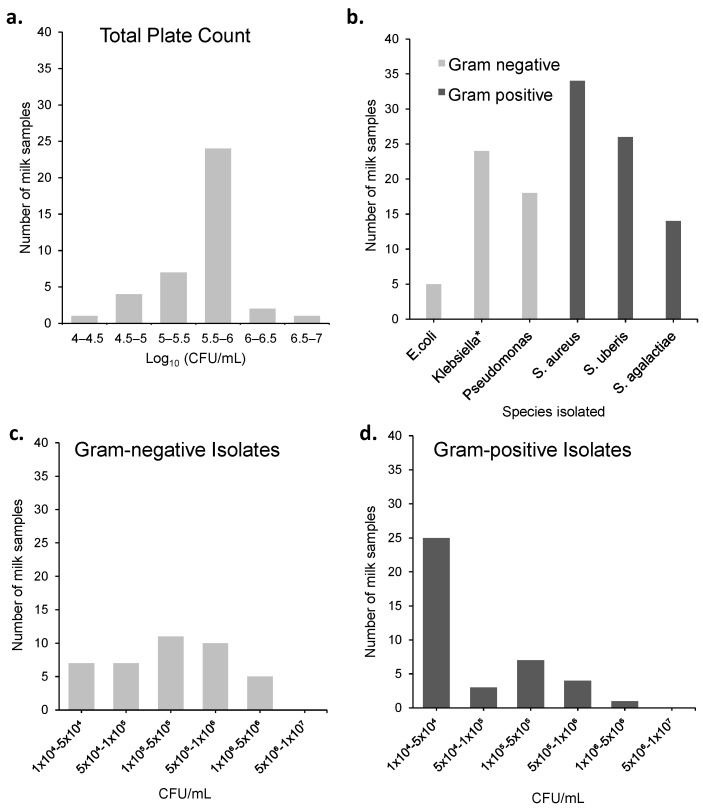
Counts in CFU/mL of bacteria isolated from 40 individual milk samples on plate count agar. (**a**) A bar chart of total plate counts from 40 individual milk samples from dairy cows. (**b**) Species of organisms isolated. * Indicates coliform species identified using CHROMagar Mastitis GN (Paris, France), presumed *Klebsiella* spp. (**c**) CFU/mL of Gram-negative organisms isolated. (**d**) CFU/mL of Gram-positive organisms isolated.

Fresh milk samples even from healthy cows have a significant bacterial load, and we first measured this to help understand how the presence of these organisms might affect rapid direct antibiotic susceptibility testing. Commensal organisms were counted and isolated from 40 normal farm milk samples from a dairy farm (CEDAR). Plate counts were as high as 10^7^ CFU/mL but with a median bacterial count of 6 × 10^5^ CFU/mL (Figure 4).

Many different species were identified indicating the broad range of organisms present, most of which can be detected with resazurin. The majority of organisms were identified as Gram-positive, with the most frequent Gram-positive being *Staphylococcus aureus*. The most abundant Gram-negative commensals were *Klebsiella* spp. and other coliforms. The least common organism across all milk samples was *E.coli*. Although Gram-positive bacteria were the most abundant across all samples, they typically had lower cell densities with the majority of samples containing 1–5 × 10^4^ Gram-positive CFU/mL. On the other hand, Gram-negative bacterial cell densities were typically between 1 × 10^5^ and 1 × 10^6^ CFU/mL.

The limit of growth detection for microcapillaries is 1 CFU [34] with a test volume of 1 microlitre; thus, if tested without dilution, all of these milk samples would eventually show detectable bacterial growth through resazurin conversion. However, the median cell densities are—as expected—somewhat lower than levels expected with clinical mastitis samples, where around 10^6^ CFU/mL appear to be typical [35]. Given that dilution is needed to avoid the milk matrix interfering with AST, it may be possible to optimize the dilution to differentiate between these different ranges of bacterial cell densities between pathogen and commensal. But as there is limited correlation between bacterial cell loads and infection, diagnosis of mastitis should not rely on bacteriological culture methods, instead clinical signs, and diagnostic tests such as the California mastitis test are more appropriate. Instead, the focus of rapid tests should be on the antibiotic susceptibility of the organisms present.

As well as considering if growth might be detected through the presence of commensal organisms found in healthy milk samples, it is critical to understand if the presence of commensals alongside pathogenic bacteria could lead to incorrect antibiotic susceptibility scores. Of particular concern is false susceptibility—where an ineffective antibiotic might be selected based on test results, leading to treatment failure and antibiotic misuse. If an AST indicates false susceptibility compared to reference standard methods, this is classified as a very major error. We predicted growth detection curves for mixed bacteria samples to determine if false susceptibility results could theoretically occur, then used simple experiments to test if this can occur in practice.

**Figure 5:**
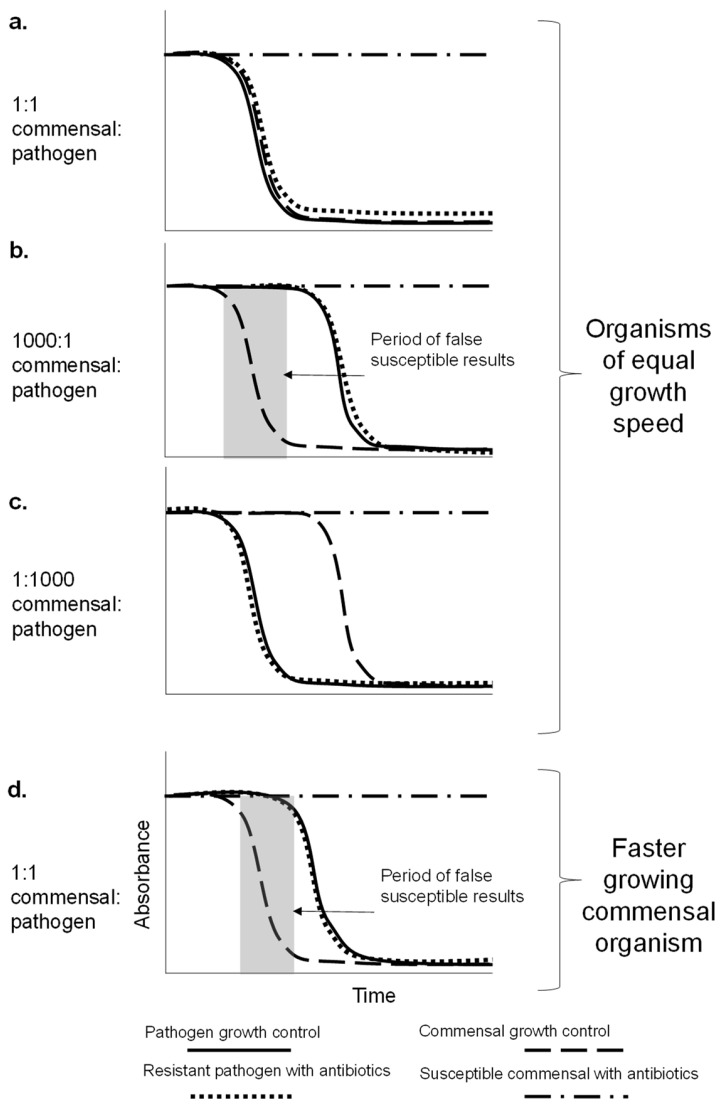
The potential problem of false susceptibility for direct antibiotic susceptibility testing of a milk sample containing a pathogen and commensal bacterial organisms. (**a**) No false susceptibility would be observed when the resistant pathogen and susceptible commensal are at the same concentration (1:1) and grow at equal speeds. (**b**) False susceptibility is observed where the susceptible commensal has a higher concentration than the resistant pathogen as no growth will be seen at early time points (1000:1 pathogen to commensal) and grow at equal speeds. (**c**) No false susceptibility resistant pathogen has a higher concentration than the susceptible commensal (1000:1 pathogen to commensal) and grow at equal speeds. (**d**) False susceptibility is observed when the resistant pathogen and susceptible commensal are at the same concentration (1:1) and the commensal is a faster-growing organism. Bacterial growth results in resazurin conversion and decrease in absorbance.

We considered theoretically what would happen in a direct test where the sample contained a fast-growing susceptible commensal organism in higher abundance than a resistant pathogen. Simulated growth curves were produced to give a visual representation of expected resazurin conversion (Figure 5). For a window of time, there could be an absence of growth detection in the presence of antibiotics during which time the test may indicate a susceptible organism; however, at later timepoints when the less abundant pathogen growth is detected, the resistance will become clear. This problem could be particularly challenging in samples where the commensal organism(s) are faster growing than the pathogen. Although it is also possible to observe false resistance with a susceptible pathogen mixed with resistant commensal, false resistance is less risky in practice, as this result would only result in avoiding the use of a potentially effective antibiotic, not lead to misuse of an ineffective treatment.

**Figure 6:**
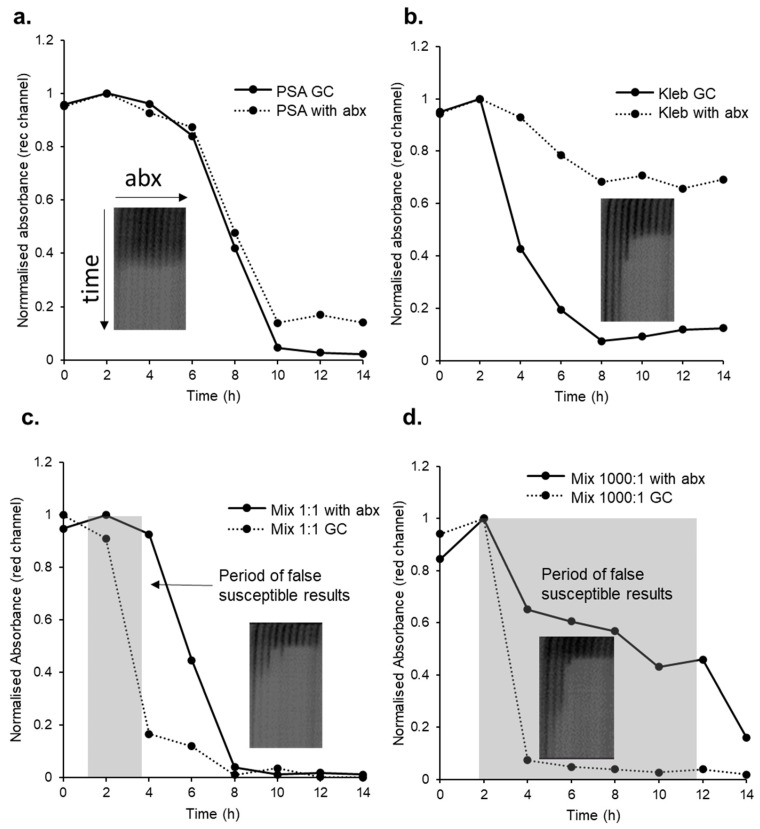
False susceptibility when a susceptible coliform (Kleb; *Klebsiella* spp.) commensal organism in is higher abundance than a resistant mastitis-causing pathogen (PSA; *Pseudomonas* spp.) in the presence of ciprofloxacin (*n* = 3). Inset images show the full MIC test strip with 9 antibiotic concentrations, capillary 1 indicates 0.5 mg/L ciprofloxacin with decreasing in log_2_ dilutions from left to right plus no antibiotic—termed growth control (GC) at the far right. Time course images indicate 18 h incubation time. Bacterial growth results in resazurin conversion and decrease in absorbance. (**a**) Growth kinetics of the resistant pathogen alone with and without ciprofloxacin at the MIC for the commensal (0.125 mg/L, capillary 3); GC indicates growth control without the presence of antibiotics. (**b**) Growth kinetics of commensal with and without ciprofloxacin at the MIC for the commensal (0.125 mg/L). (**c**) Pathogen and commensal at equal inoculum densities with and without ciprofloxacin at the MIC of the commensal (0.125 mg/L). Period of false susceptibility present. Total plate count is 8 × 10^4^ CFU/mL (**d**) Growth kinetics of the resistant pathogen spiked at 10^4^ CFU/mL and susceptible commensal at 10^7^ CFU/mL, total plate count is 3 × 10^6^ CFU/mL, with and without ciprofloxacin at the MIC of the commensal (0.125 mg/L). Period of false susceptibility present. Colour change caused by growth of the resistant pathogen in the presence of antibiotics not evident until after 10 h. abx = antibiotics.

To determine experimentally if this predicted false susceptibility can occur, we selected one resistant pathogen and one susceptible commensal isolate from farm milk samples, and mixed them at different ratios, keeping the overall cell density constant. When these simulated samples were tested using direct microcapillary AST, we plotted growth detection kinetics in the presence or absence of the minimum inhibitory concentration of antibiotic. As predicted (Figure 5) we found that if a susceptible commensal isolate is included at far high concentrations than a resistant pathogen, for a window of time false susceptibility is apparent (Figure 6). Note the organisms selected for this test have different growth rates, with a fast-growing *Klebsiella* isolate and a slower-growing *Pseudomonas* isolate. Growth rates within microcapillaries were calculated by comparing resazurin conversion times for 10-fold dilutions of each organism as described previously [34], with observed doubling times of 20.5 min (*Klebsiella*) and 34.9 min (*Pseudomonas*), which is why growth was detected later for the latter (6–8 h) than the former (2–4 h) at the same starting density (Figure 6a,b). When the two organisms are combined 1:1 (Figure 6b) there is as expected a narrow time period when false susceptibility is observed, before growth of the resistant *Klebsiella* can be detected (between 2 and 4 h, Figure 6c). The colour intensity recorded with this imaging system can drift during an experiment, as seen with resistant *Klebsiella* in the presence of antibiotics (Figure 6b) but never falls below 0.6 of starting absorbance without bacterial growth. The longest period of false susceptibility was, as predicted, seen with a far lower inoculum of resistant *Pseudomonas* than susceptible *Klebsiella* where growth was only detected at 12–14 h (Figure 6d).

## 3. Discussion

Despite the vital drive towards improved antibiotic stewardship, it has proved hard to deliver cost-effective and successful rapid AST that can inform treatment decisions by providing results fast enough to avoid days of treatment delay waiting for conventional laboratory culture methods. This is, in part, because the standard method has been highly optimized, and so changes to these methods, which might include mixed populations and/or interfering sample matrix components, can make the results more difficult to interpret. Here, we used an inexpensive microfluidic AST to examine these challenges in detail.

Direct from sample AST can potentially offer significant time saving by removing at least one overnight incubation step. Depending on the testing protocol it will also simplify sample preparation and cut handling time, offering the additional benefit of allowing tests to be performed outside of a lab, for example facilitating on-farm testing and avoiding sample transportation. We suggest that one of the simplest sample preparation options would be dilution into Mueller–Hinton broth medium. In this workflow, the dilution factor must balance reducing sample matrix interference with the uncertainty of an unknown inoculum population and density. We observed that with lower dilution factors (1:2 and 1:5) of milk or yoghurt (simulating poorly stored or spoiled sample) the colourimetric readout of resazurin was affected and growth delayed, thus further dilution may be essential to avoid sample matrix interference. It is also likely that for higher bacterial density samples, higher dilutions would be needed to avoid inoculum effects that we previously found can interfere with microfluidic AST [36]. We must also consider the potential bacteriostatic effects of lactic acid-producing *Lactobacilli* that will be particularly present in spoiled samples as in yoghurt, which by increasing membrane permeability will inhibit the growth of some Gram-negative species [37].

When considering the average bacterial cell count of a mastitis milk sample, it is difficult to find a definitive number and sample-to-sample variation is high. However, for commensal bacteria, we see legislation within the EU suggesting that ‘healthy’ raw milk can have up to 10^5^ CFU/mL before processing [38,39]. We would expect milk from an infected sample to have much higher numbers of bacteria compared to normal. However, in the case of subclinical mastitis, lower bacterial loads may remain undetected and fall within the normal bacteria microbiome found in a healthy milk sample. The standard methods for AST use an inoculum range of 2–8 × 10^5^ CFU/mL, which suggests that a mastitis sample would need to be diluted by at least 1:10 or more, reducing any risk of the milk sample matrix interfering with bacterial growth detection or susceptibility scores, given we found no effect on MIC values of milk diluted this much (Figure 2 and Figure 3).

The commensal bacteria loads that we measured in the fresh milk samples were representative of fresh uninfected milk samples. This was carried out to provide information on the commensal populations present in fresh milk. Almost all were below these limits, suggesting the abundance of these organisms is as expected. Although careful sampling of milk is a prerequisite and will help to reduce the bacterial load of commensals, differing farming practices may make sample collection for an on-site rapid test difficult to control universally. The distribution of organisms that were found in a normal healthy milk sample is quite typical with the occurrence of commensal *Streptococcus* species particularly apparent in cow’s milk [40]. Although some species detected are associated with infection, such as *E.coli*, *Staphylococcus aureus*, and *Klebsiella*, it is not uncommon for milk to become contaminated with these bacteria through contact of the udder with other areas of the dairy farm environment [41,42], manure, bedding, and other farm equipment [43]. *Klebsiella* spp. is also fast becoming one of the most common contaminants of milk samples, coming second in prevalence only to *E.coli* of Gram-negative bacteria in cow udders [43]. *Pseudomonas aeruginosa* is one of the pathogenic bacteria responsible for bovine mastitis, often with all isolates studied in one study having a genotype associated with increased SCC [44]. Although it is opportunistic and infections are infrequent, clinical mastitis caused by *Pseudomonas* species is often sporadic and severe with high rates of mortality [45,46], indicating a potential clinical significance as a pathogenic organism. Further work would benefit from the study of milk samples with both clinical and subclinical mastitis alongside healthy samples, to better understand the distribution of organisms during an infection, requiring access to a suitable number of samples. Consideration of the “resistome” of the milk microbiome during clinical mastitis is also critical [47] and it is possible that direct measurement of mixtures containing commensals plus pathogens would indicate the response to antibiotic treatment better. However, for the purpose of developing a direct test, at this early stage using a mastitis sample with a varied bacterial environment would provide conditions that are sufficiently complex that it may be too difficult to control enough to deliver robust results.

Standard AST methods require specific media, MH broth, selected to minimise interference with antibiotic activity. It is clear that changing this medium can affect observed MIC results, including pH [36,48]. Our findings indicate that dilution of milk 1:5 or 1:10 may be sufficient to reduce any impact of the milk sample matrix on growth detection and changes in MIC.

Given there is an overlap between bacterial cell densities found in mastitis samples with commensal organisms it may prove harder to avoid false positive culture from the growth of commensals found in healthy milk. If the only purpose of testing is to measure susceptibility, however, and other tests are used to diagnose infection, rapid testing should not be used to detect microorganism growth. Instead, there remains a risk of additional organisms other than the pathogen (including commensal microbiota and any contaminants during sampling) affecting the susceptibility test. We determined that a susceptible commensal would only be likely to mask the resistance of a pathogen in samples where the commensal cell density significantly exceeds the pathogen cell density.

Finally, a trade-off may be required between the endpoint time chosen, and the accuracy of results obtained using direct milk testing, with earlier readouts possibly presenting more risk of false susceptibility. Further extensive analysis of milk samples from mastitis cases is now planned with this technology, to determine how frequently such very major errors in susceptibility might occur. Moreover, further optimization of the methodology is ongoing to better understand the behaviour of Gram-positive organisms. This will be essential to ensure that Gram-positive bacteria can also be measured in milk, which is vital for application to mastitis sample testing given how common Gram-positive infections are.

## 4. Materials and Methods

### 4.1. Bacterial Isolates and Reagents

*Escherichia coli* 25922 was used as our quality control strain. *Pseudomonas aeruginosa* ATCC 12903 was used as our mock pathogen and a *Klebsiella* spp. was isolated from fresh farm milk. Ciprofloxacin and gentamicin were purchased from Sigma Aldrich (Gillingham, UK). Pasteurised supermarket milk from The Cooperative Food Company (Manchester, UK) was chosen to standardise experiments, and because it has a low bacterial load following processing. Yeo Valley natural yoghurt was purchased from The Cooperative Food Company. Individual milk samples were collected from healthy cows from the Centre for Dairy Research (CEDAR) (Reading, UK) farm (n = 40). Milk samples were tapped off of the automated milking parlour system into sterile collecting pots and labelled according to cow number. Milk samples were immediately transported back to the microbiology lab (~15 mins transportation time).

### 4.2. Preparation of Antibiotic Microcapillary Dip-Stick Test Strips

Microcapillary antibiotic test strips were prepared as previously described [31]. Briefly, MCF of 1 m lengths were coated internally with a hydrophilic layer of 5 mg mL^−1^ solution of polyvinyl alcohol (PVOH, MW 146,000–186,000, >99% hydrolysed, Sigma-Aldrich) in water and incubated at room temperature for 2 h. Coated strips were washed with 0.5% Tween 20 in water (Sigma-Aldrich, UK) to remove residual PVOH, and dried on a vacuum manifold for 20 min. Ciprofloxacin and gentamicin antibiotic stock solutions were prepared. Final concentrations of antibiotics indicated in the text, diluted in sterile Mili-Q water, were injected into individual capillaries using a sterile 30 G needle. The MCF was cut into 17 mm individual test strips and frozen overnight at −80 °C. Test strips were freeze-dried for >4 h on an Edwards Modulyo freeze drier. Test strips were placed into custom 3D-printed holders and were vacuum packed and stored at −20 °C until use.

### 4.3. Microcapillary Antibiotic Susceptibility Test

Bacterial strains were routinely grown from glycerol stocks overnight on LB agar. Approximately 4–5 colonies were grown in Mueller–Hinton (MH) broth for several hours until turbid and adjusted to 0.5 McFarland standard, according to CLSI guidelines. Bacterial suspensions were diluted a further 1:150 in MH broth and 100 µL of bacterial suspension was added to 100 µL 0.5 mg/mL resazurin in MH broth to give a final concentration of 0.25 mg/mL resazurin and 5 × 10^5^ CFU/mL bacteria in a 96 well plate. Microcapillary test strips were dipped into the wells and the strips sealed with silicone grease to avoid evaporation. Samples were incubated at 37 °C overnight, colour change was time-lapse imaged using Raspberry Pi-operated automated imaging devices PiRamid [33]. The MIC was determined based on the lowest concentration that inhibited the reduction in resazurin from blue to pink after overnight incubation. 

The effect of different milk samples on assay results was also studied. The antibiotic susceptibility test was performed as before with the addition of pasteurized milk or natural yoghurt, to simulate changes in spoiled milk. The concentrations of milk/yoghurt were prepared: 100%, 40%, 20%, 10%, and 2%. Then, 100 μL of bacteria solution and 100 μL of each milk dilution were pipetted into rows of a 96-well plate, with a final resazurin concentration of 0.25 mg/mL. The final estimated cell density of *E. coli* was CFU/mL of 5 × 10^5^ and dilutions of milk were 50%, 20%, 10%, 5%, and 1%, with a MH broth control.

MIC measurements were taken in duplicate with two antibiotic-coated strips per dilution of milk or yoghurt. The MIC was recorded as the lowest concentration of antibiotic that did not show resazurin conversion in the capillaries. For tests that varied in duplicate measurements, the highest MIC was recorded. A growth control (no antibiotic) capillary was included for all test strips.

### 4.4. Total Plate Count and Bacterial Identification of Fresh Milk Samples from Healthy Cows

Milk samples from healthy cows collected from CEDARs were diluted 1:100 in 9% saline solution and 10 µL was pipetted and spread onto two individual sterile plate count agar plates for cell enumeration (Sigma-Aldrich, UK). For presumptive identification, 10 µL of each milk sample was pipetted and spread onto a single CHROMagar Mastitis GN and GP agar plate for identification of species of bacteria (CHROMagar™ Mastitis). CFU/mL was calculated for each sample after overnight incubation on plate count agar. CHROMagar plates were examined, and species present were confirmed by colour scoring of visible colonies, according to CHROMagar (CHROMagar™ Mastitis).

### 4.5. MIC Determination in Simulated Mastitis Samples with Mixed Bacterial Populations

Antibiotic strips containing ciprofloxacin were prepared as above with 2-fold dilutions from a maximum concentration of 0.5 mg/mL. MIC for bacterial species *Pseudomonas aeruginosa* ATCC 12903 and a *Klebsiella* spp. were performed with a total bacterial density of 5 × 10^5^ CFU/mL and varying mixtures of the two were determined as indicated in the text. These represented a potential mastitis sample that contained both pathogen plus non-pathogenic commensal (or contaminant). Bacterial density was adjusted based on turbidity, with 0.5 McFarland estimated at 10^8^ CFU/mL and confirmed by overnight plate counts. Bacteria were diluted in MH broth and resazurin at a final concentration of 0.13 mg/mL. Ciprofloxacin-loaded MCF strips were dipped into each of the wells and end covers filled with silicone grease were placed on each end to stop sample evaporation. Samples were incubated overnight (20 ± 4 h) at 37 °C and colour change was time-lapse imaged using PiRamid. Time-lapse data were retrieved to evidence the difference in growth time between species and inoculum densities and demonstrate occurrences of false susceptibility.

### 4.6. Imaging and Image Analysis

All time-lapse imaging was carried out on a low-cost open-source imaging system designed for time-lapse imaging of colourimetric assays [33]. Image J was used to analyse the time-lapse image series of bacterial growth in the microcapillary test strips. Colour images were split into blue, blue and green (RGB) channels and the red channel was chosen for intensity. A line across the centre of each test strip was highlighted and re-sliced across an image stack of all timepoints to produce a composite image with the x-axis representing position across the strip of 10 capillaries, and a vertical axis representing incubation time. Absorbance (*A*) values at chosen timepoints were calculated with the sample (*I*) and background (*I_o_*) intensity values using:

*A* = *log*_10_ (*I_o_*/*I*)

The time for resazurin conversion was calculated based on the image timepoint when resazurin absorbance reached a threshold conversion: (mean signal for no bacteria absorbance) + 3 × (standard deviation of control absorbance).

## Data Availability

All data supporting the findings of this study are presented within the paper.
